# Dysphonia in adults with developmental stuttering: A descriptive study

**DOI:** 10.4102/sajcd.v64i1.347

**Published:** 2017-06-26

**Authors:** Anél Botha, Elizbé Ras, Shabnam Abdoola, Jeannie van der Linde

**Affiliations:** 1 Department of Speech-Language Pathology and Audiology, University of Pretoria, South Africa

## Abstract

**Background:**

Persons with stuttering (PWS) often present with other co-occurring conditions. The World Health Organization’s (WHO) International Classification of Functioning, Disability and Health (ICF) proposes that it is important to understand the full burden of a health condition. A few studies have explored voice problems among PWS, and the characteristics of voices of PWS are relatively unknown. The importance of conducting future research has been emphasised.

**Objectives:**

This study aimed to describe the vocal characteristics of PWS.

**Method:**

Acoustic and perceptual data were collected during a comprehensive voice assessment. The severity of stuttering was also determined. Correlations between the stuttering severity instrument (SSI) and the acoustic measurements were evaluated to determine the significance. Twenty participants were tested for this study.

**Result:**

Only two participants (10%) obtained a positive Dysphonia Severity Index (DSI) score of 1.6 or higher, indicating that no dysphonia was present, while 90% of participants (*n* = 18) scored lower than 1.6, indicating that those participants presented with dysphonia. Some participants presented with weakness (asthenia) of voice (35%), while 65% presented with a slightly strained voice quality. Moderately positive correlations between breathiness and SSI (*r* = 0.40, *p* = 0.08) have been reported. In addition, participants with high SSI scores also scored a poor DSI of below 1.6, as observed by a moderate positive correlation between SSI and DSI (*r* = 0.41).

**Conclusion:**

The majority of PWS presented with dysphonia, evident in the perceptual or acoustic parameters of their voices. These results can be used for further investigation to create awareness and to establish intervention strategies for voice disorders among PWS.

## Introduction

Developmental stuttering (DS) is the most common type of stuttering, affecting approximately 1% of the adult population (Watkins, Smith, Davis & Howell, 2008). It is also a complex and multifactorial disorder as many different factors play a role (Guitar, 2014). More specifically DS is related to abnormalities within the speech planning and production and auditory feedback during speech (Ingham et al., 2004). During stuttering, there is abnormal functioning of the whole speech system, including the larynx (Salihović, Junuzović-Žunić, Ibrahimagić & Beganović, 2009). Abnormal functioning of the larynx may include excessive muscular tension and variable subglottal pressure, which could be caused by muscle incoordination of the respiratory tract. Weaker laryngeal neuromuscular control and disturbances in respiratory and laryngeal control may also lead to voice problems (Salihović et al., 2009).

Persons with stuttering (PWS) often present with other co-occurring conditions such as anxiety, a low self-esteem and negative responses to their communication partners (Erickson & Block, 2013). These co-occurring conditions may complicate the communication process and affect the emotional well-being and quality of life of PWS negatively (Blood, Blood, Maloney, Meyer & Qualls, 2007). An individual’s feelings and attitudes can be as much part of the disorder of stuttering as his speech behaviours. At first, stuttering may result in unnoticeable, repetitive stuttering behaviours. As stuttering increases, feelings of frustration and shame may also increase, which result in tense and effortful speech that impede fluency (Guitar, 2014). PWS may have negative attitudes about themselves, which are derived from years of stuttering experiences, and they often project these attitudes on listeners. The relationship between stuttering and emotions varies among individuals who stutter. For some individuals, emotions may contribute to the aetiology of stuttering and for others, stuttering may evoke emotions such as frustration, fear and anger (Guitar, 2014).

A basic and powerful way to express one’s emotions and to convey messages is through the voice (Frühholz, Trost & Grandjean, 2014). The human voice has been described as an embodiment of self in a social context, contributing to expression, perception and mutual exchange of self, consciousness, inner life and personhood (Sidtis & Kreiman, 2011).

If PWS present with negative emotions or anxiety as a result of different factors, including stuttering, it may negatively impact the voice characteristics, such as voice quality (Pullin & Cook, 2013). Voice problems in PWS may occur because of attempts to mask their stuttering by changing their pitch or volume to an inappropriate level, leading to a misuse in voice (Cooper, 1979). Yet, only Cooper (1979) and Salihović et al. (2009) have conducted studies to explore the voice quality of PWS. However, few studies have explored the relationship between dysphonia and PWS in the past.

The World Health Organization’s (WHO) International Classification of Functioning, Disability and Health (ICF) proposes that it is important to understand the full burden of a health condition. To determine the impact of the condition, information about the disorder and how it impacts the functioning of an individual needs to be determined. Because only a few studies have explored voice problems in PWS, the importance of conducting future research has been emphasised (Salihović et al., 2009). From the gap in existing literature and the multimodality of stuttering, as well as the effect of stuttering on all the different areas of an individual’s life, the following research question is posed: *What are the vocal characteristics of adults diagnosed with a developmental stutter?*

## Methods

### Aim

The study aimed to describe the vocal characteristics of adults with DS.

### Setting and participants

The study was conducted at the Department of Speech-Language Pathology and Audiology, University of Pretoria. A total of 20 adults who were able to speak either Afrikaans or English as first or second language were selected to take part in the study. Inclusion criteria required the participants to exhibit with a DS only, with no other co-occurring communication disorders. Individuals who had any known speech, language, hearing disorders or syndromes, apart from stuttering, were excluded from the study. The participants had to be between the age of 18 and 60 years. Anatomical and physiological changes of the vocal folds occur as individuals age and affect the acoustic output of the voice (Xue & Deliyski, 2001). Therefore, participants over the age of 60 were excluded from this study. Participants also had to be non-smokers as smoking has an evident effect on some acoustic voice parameters (Banjara, Mungutwar, Singh & Gupta, 2014).

### Description of participants

Case histories indicated that all 20 participants were diagnosed with DS during their preschool years and have previously received or are still receiving stuttering therapy. None of the participants have been diagnosed with a voice disorder or are receiving voice therapy. The study included 7 (35%) women and 13 (65%) men. The average age of participants were 23.5 years (s.d. = 4.95). The home language distribution included 30% (*n* = 6) English, 25% (*n* = 5) Afrikaans, 20% (*n* = 4) Sepedi, 10% (*n* = 2) Setswana and isiZulu and 5% (*n* = 1) Sesotho speakers. Approximately a third (35%) of the participants reported to have an average daily intake of carbonated drinks of one to two cups per day and 10% an average intake of three to four cups per day. A few participants (10%) reported an alcohol consumption of one to two glasses per week, and only 5% indicated that they consume three to four glasses per week. Only one participant reported to take anti-histamines for allergy-related conditions, and one participant Zalascopyrin and Celebrex for juvenile arthritis. Five participants (25%) indicated that they regularly experienced symptoms of sinusitis. One participant (5%) reported endocrine illness and one (5%) presented with oesophageal reflux. Participant 4 and Participant 19 indicated that they had experienced a change in their voice quality over time, with Participant 4 experiencing a weakness in the voice and Participant 19 experiencing hoarseness. However, no voice problems were diagnosed in any of the participants, and they were not receiving treatment for their voice symptoms.

### Voice and speech assessment protocol

#### Background questionnaire

The background questionnaire consisted of questions about the participant’s background history as well as questions related to their voice and stuttering experiences. Demographic information such as the participant’s language, race and marital status was also obtained through the questionnaire. The questionnaire used in the study by Van Wyk et al. (2016) was used as a guide to the questions used.

#### Acoustic voice analysis

The Computerized Speech Lab 4300 hardware system was used for the analysis and feedback of acoustic measurements. A comprehensive voice analysis was conducted by using the Multi-Dimensional Voice Program and the Voice Range Profile software packages (KayPENTAX, 2008) to evaluate the voice quality of the participants. The parameters that were assessed included the jitter, shimmer, highest frequency, lowest intensity, noise-to-harmonics ratio and the fundamental frequency values of a phonated/a/sound (Table 1). The Voice Range Profile displays the vocal intensity range versus fundamental frequency (F0).

**TABLE 1:** Description of acoustic parameters.

Vocal parameters	Description
Highest and lowest intensity	Degree of vocal fold excursion (Ferrand, 2012)
Highest and lowest frequency	Complete range of possible F0s a person can generate (Ferrand, 2012)
Jitter (%)	Cycle-to-cycle frequency variability (Ferrand, 2012)
Shimmer (%)	Cycle-to-cycle amplitude variability (Ferrand, 2012)
MPT	Can be referred to as the longest possible phonation of the/a/sound (measured in milliseconds) after maximum inspiration, given two attempts (Maslan, Leng, Rees, Blalock & Butler, 2011)
S–Z ratio	The S–Z ratio is a measure of laryngeal pathology. It measures the length of time a person can sustain the/s/sound and the/z/sound. The two figures are then divided to acquire a numerical ratio. The higher the figure, the greater the possibility that the person experiences difficulty with phonation (Eckel & Boone, 1981)

MPT, maximum phonation time.

The Dysphonia Severity Index (DSI) is an index used to create an objective and quantitative link of the perceived voice quality. The DSI is an objective measure as no perceptual analysis is necessary to determine the index. The DSI ranges from +5 (i.e. no dysphonia are present) to -5 (i.e. a severely dysphonic voice). The smaller the DSI, the greater the severity of the dysphonia. While normal voice quality is evident when a positive DSI (1.6 and higher) is obtained (Wuyts et al., 2000), the voice measurements that are used to calculate the DSI include the lowest intensity (I-Low in dB), highest frequency (F0-High in Hz), maximum phonation time (MPT in seconds) and the jitter (%), (see Equation 1):

$$\begin{array}{l} \text{DSI}=0.13\times \text{MPT}+0.0053\times \text{F}0-\text{High}-0.26\times \\ 1-\text{Low}-1.18\times \text{Jitter}(\%)+12.4 \end{array}$$ [Eqn 1]

#### Perceptual voice and speech analysis

The perceptual voice and speech analysis of the clients was conducted through the analysis of a spontaneous speech sample and a recorded speech sample of the participants’ voices. The recordings were obtained through the participants who read either an Afrikaans or English passage [*Na die Wildtuin/Rainbow passage* (Fairbanks, 1960)]. The instruments that were used included the grade of hoarseness, roughness, breathiness, asthenia, strain and instability (GRBASI) 4-point scale (Yamauchi, Imaizumi, Maruyama & Haji, 2010) and the stuttering severity instrument (SSI-4; Riley, 2009).

The GRBASI perceptual rating scale was recommended by the Japanese Society of Logopedics and Phoniatrics and the European Research Group for the use in clinical and research settings (Yamauchi et al., 2010). The SSI-4 was used to determine the frequency, duration, physical concomitants and the severity of stuttering. Based on these parameters, the SSI was determined as very mild, mild, moderate, severe or very severe (Riley, 2009). A speech sample that was recorded during the voice assessment was used to determine the severity of stuttering. The ratings were performed after the voice assessment.

A listener panel, consisting of three listeners, took part in the perceptual analysis. To increase the reliability and validity of the perceptual analysis, a blind rating was performed by the three listeners. All three listeners had normal hearing and were student researchers.

#### Voice Handicap Index

The Voice Handicap Index (VHI) measures the influence of voice problems on a patient’s quality of life. (Maertens & de Jong, 2007). The VHI is a 30-item self-administered questionnaire (subjective rating scale) that required from the participants to describe their voice and the effects of their voice on their life. The VHI consists of three subscales, which cover the areas of functional, emotional and physical aspects of voice disorders. The VHI was scored after the assessment by the researchers. The overall score was interpreted as mild, moderate or severe.

**Data analysis:** All data were analysed by means of descriptive statistics by using statistical software (STATA). Correlations between the SSI and the acoustic measurements, including the strength and direction (negative or positive) of a relationship between two variables, were evaluated using Spearman’s rank correlation coefficients. Correlations between the variables of 0.2 ≤ 0.39 were classified weak; 0.4 ≤ 0.59 were moderate; 0.6 ≤ 0.79 were strong; and 0.8 ≤ 1.0 were very strong (Mukaka, 2012). Pearson’s correlation coefficients were calculated between normal distributed variables, and the non-parametric Spearman’s rank correlation coefficients for correlation between the non-normal variables.

Spearman’s rank and, where appropriate, point bi-serial correlation values were determined between SSI, DSI, VHI and the demographic variables. Chi-squared tests were used to identify significant associations at 5% and 10% significance level between the categorical demographic variables, GRBASI scores and SSI categories.

## Results

The stuttering severity of participants (*n* = 20) is depicted in Table 2. The majority, 75% (*n* = 15), of participants presented with a very mild stutter, while only 10% (*n* = 2) presented with a severe stutter. The type of stuttering observed in participants consisted of syllable repetitions, 70% (*n* = 14), prolongations, 55% (*n* = 11), blocks, 55% (*n* = 11), and avoidance of words, 40% (*n* = 8). The estimated duration of the blocks in 40% (*n* = 8) of the participants was between 2 and 9 s, one full second in 25% (*n* = 5) of the participants and the remaining 35% (*n* = 7) presented with estimated duration of less than 1 s. No physical concomitants were observed in 65% (*n* = 13) of participants during moments of stuttering. However, very distracting sounds were observed in 15% (*n* = 3) of participants, distracting facial grimaces in 20% (*n* = 4) of participants and very distracting head movements in 20% (*n* = 4) of the participants.

**TABLE 2:** Description of stuttering severity of participants (*n* = 20) according to the stuttering severity instrument.

Stuttering severity	*n*	%
Very mild	15	75
Mild	2	10
Moderate	1	5
Severe	2	10
Very severe	0	0

The vocal characteristics of the participants are presented according to perceptual analysis of voice, acoustic voice analysis and self-rating on the VHI. The consensus score across the GRBASI scale (Table 3) indicated that the perceptual results of many of the participants deviate from normal voice quality. Some participants presented with a slight weakness (asthenia) of voice (35%), while the majority of participants (65%) presented with a slightly strained voice quality.

**TABLE 3:** Consensus scores of participants (*n* = 20) across grade of hoarseness, roughness, breathiness, asthenia, strain and instability scale.

Consensus score	Grade		Roughness		Breathiness		Asthenia		Strain		Instability	
	*n*	%	*n*	%	*n*	%	*n*	%	*n*	%	*n*	%
0 (normal)	11	55	11	55	15	75	13	65	5	25	17	85
1 (slight)	7	35	9	45	5	25	7	35	13	65	3	15
2 (moderate)	2	10	0	-	0	-	0	-	2	-	0	-
3 (severe)	0	-	0	-	0	-	0	-	0	-	0	-

In Table 4 the results of the acoustic voice analysis are presented. The median and interquartile ranges (IQRs) are compared to the expected norms for each acoustic outcome and the correlations between them.

**TABLE 4:** Acoustic analysis: Descriptive statistics and norms.

*p* (Wilcoxon sign test statistic)	Median (*n* = 20)	IQR	Norms (Ferrand, 2012)	*p*-value (Wilcoxon sign test statistic)
Jitter	0.014	0.006–0.208	0.2% – 1.0%	< 0.001*
Shimmer	0.052	0.031–0.107	Less than 5%	0.588
Maximum frequency	392.65	277.65–570.85	700–1000 Hz	< 0.001*
Minimum frequency	100.92	82.41–123.47	80–135 Hz	0.503
Maximum intensity (dB)	97.5	89.0–103.0	100–110 dB	0.252
Minimum intensity (dB)	64.5	62.0–67.0	40 dB	< 0.001*
s–z ratio	0.81	0.68–1.24	1.0–1.4	0.132
Maximum/a/-phonation time	13.16	8.63–16.54	20 s	0.001*
DSI	−2.3	−3.6–0.25	1.6 or higher	< 0.001*

IQR, interquartile ranges; dB, decibel; DSI, Dysphonia Severity Index.

* , *p* ≤ 0.05.

The jitter (*p* < 0.001), maximum frequency (*p* < 0.001), minimum intensity (*p* < 0.001), MPT (*p* = 0.001) and the DSI (*p* < 0.001) all presented with significant differences between the recorded medians and the norms. The shimmer, minimum frequency, maximum intensity and the s–z ratio are the only measurements whose medians fall within the norm or are relatively close to the norm. The median minimum intensity (65 dB) is significantly louder than the norm of 40 dB. The MPT is significantly lower than the norm of 20 s, indicating that most participants were not able to phonate the/a/sound for 20 s or more. Only 15% of the participants were able to sustain phonation of the/a/sound for 20 s or more. The DSI results also indicated a significantly lower score when compared to the norm, indicating a moderate dysphonia in the participants. Only two participants (10%) obtained a DSI score of 1.6 or higher while 90% (*n* = 18) of participants scored lower than 1.6.

According to the self-rating of the VHI, 40% (*n* = 8) of the participants scored between 0 and 30 on the VHI, indicating a low level score. The majority of participants, 55% (*n* = 11), scored between 31 and 60, indicating a moderate level of handicap and only 5% (*n* = 1) greater than 60 points, indicating a severe level of handicap. The statements shown in Table 5 were extracted from the VHI and represent the statements that received the highest scores from participants.

**TABLE 5:** Voice Handicap Index statements with highest self-rating scores by participants (*n* = 20).

Statements	Never		Almost never		Sometimes		Almost always		Always	
	*n*	%	*n*	%	*n*	%	*n*	%	*n*	%
I use the phone less often than I would like to.	4	20	2	10	8	40	4	20	2	10
I run out of air when I talk.	1	5	2	10	8	40	7	35	2	10
I use a great deal of effort to speak.	3	15	7	35	5	25	5	25	0	-
The sound of my voice varies throughout the day.	5	25	4	20	7	35	2	10	2	10

In this study, participants with high SSI scores, also scored a poor DSI of below 1.6, as observed by a moderate positive correlation between SSI and DSI (*r* = 0.41).

Although the correlations given in Table 6 are not all statistically significant, the directions of the correlations are of clinical significance. The jitter (*r* = -0.62) displayed a moderate to strong negative correlation with the DSI, implying that participants who scored poorly on this parameter also scored lower than 1.6 on the DSI. In contrast, maximum frequency (*r* = 0.40) showed a positive relationship with the DSI, indicating that participants with a maximum frequency showed lower than the norm also scored lower than 1.6 on the DSI.

**TABLE 6:** Acoustic outcomes and correlations between the stuttering severity instrument, Dysphonia Severity Index and Voice Handicap Index and acoustic variables.

Variable	SSI		DSI		VHI	
	*r*	*p*	*R*	*P*	*r*	*p*
SSI	1.00	-	-	-	-	-
DSI	0.41	0.08*	1.00	-	-	-
VHI	−0.04	0.86	−0.26	0.27	1.00	
Jitter	−0.28	0.24	−0.62	0.57	0.00	0.99
Shimmer	−0.08	0.75	−0.37	0.11	−0.36	0.12
Voice fundamental frequency variation	−0.12	0.61	−0.65	0.35	−0.20	0.40
Maximum frequency	−0.09	0.72	0.40	0.08*	−0.10	0.67
Minimum frequency	−0.36	0.12	0.15	0.53	−0.26	0.27
Maximum intensity (dB)	−0.11	0.65	0.08	0.74	0.32	0.16
Minimum intensity (dB)	−0.54	0.01**	−0.19	0.42	0.12	0.62
s–z ratio	−0.16	0.50	0.01	0.97	−0.29	0.22
Maximum/a/-phonation time	0.08	0.73	0.33	0.15	−0.13	0.59

SSI, stuttering severity instrument; DSI, Dysphonia Severity Index; VHI, Voice Handicap Index; dB, decibel.

* , *p* ≤ 0.1

** , *p* ≤ 0.05.

Spearman’s rank correlation coefficients were calculated for the GRBASI scales and SSI, DSI, VHI and participant characteristics (Table 7). Moderate positive correlation was observed between breathiness and SSI (*r* = 0.40; *p* = 0.08). A moderate positive correlation between oesophageal reflux and VHI was noted (*r* = 0.45; *p* = 0.05), indicating that the participant with oesophageal reflux scored higher on the VHI. Significant differences were observed between home language and the asthenia score (*p* = 0.024). It appears that Sepedi and Setswana speakers obtained a weaker rating of 1, compared to English and Afrikaans speakers who were rated 0 for asthenia. Further significant associations were observed between the strain score and weekly alcohol consumption (*p* = 0.042), as well as daily consumption of carbonated drinks (*p* = 0.022). The higher the daily consumption of alcohol and carbonated drinks, the higher is the perceived strain in voicing. No other significant associations between the perceptual evaluations and gender, marital status, medical history and excessive shouting were observed in this study.

**TABLE 7:** Correlations between the stuttering severity instrument, Dysphonia Severity Index and Voice Handicap Index and grade of hoarseness, roughness, breathiness, asthenia, strain and instability scale.

Variable	SSI		DSI		VHI	
	*r*	*p*	*r*	*P*	*r*	*p*
Grade	0.10	0.69	−0.02	0.94	0.11	0.63
Roughness	−0.01	0.97	−0.32	0.17	−0.17	0.49
Breathiness	0.40	0.08*	0.06	0.80	0.06	0.80
Asthenia	0.28	0.23	0.17	0.47	0.35	0.14*
Strain	0.24	0.30	0.01	0.98	−0.17	0.48
Instability	0.15	0.54	−0.19	0.41	0.01	0.96

SSI, stuttering severity instrument; DSI, Dysphonia Severity Index; VHI, Voice Handicap Index.

* , *p* ≤ 0.1.

## Ethical considerations

Ethical clearance was obtained from the Research Ethics Committee, Department Speech Language Pathology and Audiology, University of Pretoria, prior to data collection, with ethics clearance number: 2016/13024753/04498063/13042964. Once written informed consent was provided, the comprehensive voice and speech assessment protocol was conducted.

## Discussion

The severity of DS in the current study ranged between very mild to moderate. Supporting the outcomes of the current study, a previous study reported a mean stuttering severity in the mild to moderate range (Blumgart, Craig & Tran, 2010).

The perceptual analysis of voice has shown moderately positive correlations between breathiness and SSI (*p* = 0.08). During the reading activity and spontaneous speech sample, 25% of the participants displayed a breathy voice quality. A breathy voice is characterised by air loss through loosely or hypo-adducted vocal folds (Ferrand, 2012). Breathiness is often accompanied by low vocal intensity and a lower than optimum fundamental frequency level (Ferrand, 2012). Speech-language therapists often recommend the use of the easy voice onset technique to PWS. If PWS utilise this technique, they will not adduct their vocal folds as forcefully; therefore, an increased breathy voice quality may be audible (Adler, Hirsch & Mordaunt, 2006).

Interestingly, the acoustic analysis of voice rendered significantly different mean values for jitter (*p* ≤ 0.001), maximum frequency (*p* ≤ 0.001), minimum intensity (*p* ≤ 0.001), MPT (*p* = 0.001) and the DSI (*p* ≤ 0.001) when compared to the norms. The study also indicated that a higher SSI score resulted in a higher DSI score (*r* = 0.41, *p* = 0.08). Most participants were not able to sustain voicing for 20 s or more. MPT is often reduced in individuals with dysphonia (Ferrand, 2012). The reduced MPT may emphasise the fact that the PWS may struggle with prolongations of sounds and present with more dysphonic voices (Bogaardt, Speyer & Zumach, 2008). Reduced MPT also indicates decreased efficiency of the respiratory mechanism during phonation, causing inefficient use of air in PWS (Bogaardt et al., 2008). An interesting and unexpected finding was the significant difference between the norms and maximum frequency and minimum intensity and this should be explored in future research.

Poor jitter values were associated with a poorer DSI and higher VHI score. The jitter may be affected mainly because of lack of control of vocal fold vibration, in the moments of stuttering which may result in the presence of noise at emission and breathiness of the voice (Amaro, Schreiber & Wertzner, 2005).

Participants who scored higher in the VHI also had a higher asthenia rating in the GRBASI scale. Asthenia refers to the degree of weakness of the voice. The weakness of the voice can be caused by an overuse of voice or using the voice ineffectively (Ferrand, 2012). The PWS may change the way they use their voice to compensate for the emotions, anxiety and stuttering symptoms experienced (Guitar, 2014).

The demographic variances have shown some significant differences between home language and the asthenia score (*p* = 0.024). It appears that Sepedi and Setswana speakers obtained a weaker rating of 1, compared to English and Afrikaans speakers who were rated 0 for asthenia. A study by Boyer and Zsiga (2013) has indicated that depending on the geographical area, many of the Setswana speakers who participated in their study reported to have post-nasal devoicing. The devoicing of some sounds in the African languages may have contributed to the weaker asthenia ratings. Devoicing of sounds might also be interpreted as a weak voice (Pinho, Jesus & Barney, 2009).

A noteworthy association between a higher daily consumption of alcohol (*p* = 0.042) and carbonated drinks (*p* = 0.022) and higher perceived strain in voicing, was found. The use of excessive alcohol causes dehydration of the vocal folds and may also lead to irritation of the mucous membranes that line the throat (Ferrand, 2012). A study by Baek and Bae (2013a, 2013b) also stated that the use of alcohol can reduce the flexibility and elasticity of the mucosa tissue of the vocal cords. As a result incomplete opening or closure of the glottis reduces clarity of the speaker’s pronunciation because of the air leak when speaking after drinking (Geumran & Muyungjin, 2013).

This study is one of few studies that investigated the vocal characteristics of PWS. This explorative study was conducted to identify whether an experimental study is warranted. It is therefore recommended that future research should use an experimental research design on a larger sample size, including a control group that are age and gender matched. Further research should also be conducted in the paediatric population as a means to prevent voice disorders in PWS by creating awareness and establishing good vocal habits.

## Conclusion

A few studies have explored voice problems in PWS, and the characteristics of voices of PWS are relatively unknown. The WHO’s ICF proposes that it is important to understand the full burden of a health condition. Because of limited research in this area and the importance and extent to which stuttering affects a person, the aim was to describe the acoustic and perceptual parameters of voice quality in adults with DS. Results obtained from this study indicated that a higher SSI score resulted in a poorer DSI score. It has been found in the current study that the majority of PWS presented with dysphonia. Further investigation is warranted to establish prevention and intervention of voice disorders in PWS.
